# Association between non-dipping blood pressure pattern and different glucometabolic profile during oral glucose tolerance test

**DOI:** 10.1007/s11739-023-03442-1

**Published:** 2023-10-06

**Authors:** Valentino Condoleo, Raffaele Maio, Velia Cassano, Leonilde Bonfrate, Corrado Pelaia, Giuseppe Armentaro, Sofia Miceli, Teresa Vanessa Fiorentino, Maria Perticone, Elena Succurro, Francesco Andreozzi, Giorgio Sesti, Angela Sciacqua

**Affiliations:** 1 Department of Medical and Surgical Sciences, University Magna Græcia of Catanzaro, Campus Universitario “S. Venuta”, Viale Europa—Località Germaneto, 88100 Catanzaro, Italy; 2 Department of Biomedical Sciences and Human Oncology, University of Bari Aldo Moro, Bari, Italy; 3 Research Center for the Prevention and Treatment of Metabolic Diseases (CR METDIS), University Magna Græcia, 88100 Catanzaro, Italy; 4 Department of Clinical and Molecular Medicine, Sapienza University of Rome, Rome, Italy

**Keywords:** Blood pressure, Diabetes, Hypertension, Insulin resistance

## Abstract

It is known that, a not physiological blood pressure (BP) circadian pattern has been associated with increased risk of organ damage and cardiovascular (CV) event. The aim of this study was to assess the association between circadian BP pattern and glucometabolic phenotypes occurring after oral glucose tolerance test (OGTT). We recruited 810 hypertensive Caucasian patients. All participants underwent to OGTT, laboratory test and 24-h ambulatory BP monitoring (ABPM). The analysis of collected data allowed classifying patients based on nocturnal BP profiles into four categories: dippers, non-dippers, extreme dippers, and reverse dippers. Considering the dipping pattern, the proportion of non-dippers in normal glucose tolerance patients with 1-h glucose ≥ 155 mg/dL (NGT ≥ 155) (36.4%) was higher than NGT < 155 (29.6%) and impaired glucose tolerance (IGT) (34.8%), but lower than type 2 diabetes group (T2DM) (52.6%) (*p* = 0.001). The proportion of dippers was lower in NGT ≥ 155 (47%) and T2DM (34.6%), when compared with NGT < 155 (53.8%) and IGT (51.2%) (*p* = 0.017). From logistic regression analysis, 1-h glucose ≥ 155 increased the risk of a pathological nocturnal drop in BP by 74%, (OR = 1.740, 95% CI 1.254–2.415, p < 0.0001). In addition, the improvement in 1 unit of Matsuda was responsible for a 3.5% risk decrease (OR = 0.965, 95% CI 0.958–0.971, *p* < 0.0001), while e-GFR determined a 0.9% risk reduction of nocturnal BP drop (OR = 0.991, 95% CI 0.984–0.999, *p* = 0.020). Our data demonstrated the existence, in newly diagnosed hypertensive patients, of an association between circadian BP profile and altered glycemic response during OGTT, in particular NGT ≥ 155 subjects are associated with a non-dipper BP pattern, this is clinically relevant because may explain, at least in part, the increased CV risk in this setting of patients.

## Introduction

Arterial hypertension is a widespread disease with a prevalence in the adult population of 30–45%, which increases with age, thus affecting more than 60% of elderly people [1, 2]. The neuroendocrine system is significantly implicated in the circadian regulation of blood pressure (BP). In this regard, multiple interactions occur among the components of complex networks including monoaminergic, hypothalamic–pituitary–adrenal, hypothalamic–pituitary–thyroid, opioid, and renin–angiotensin–aldosterone systems (RAAS), as well as endothelial-derived vasoactive mediators [3]. Such mechanisms contribute to explain, at least in part, BP variability (BPV), characterized by continuous changes detectable within the context of very short, short and long periods. The short-term variations, involving daily oscillations that can be easily detected through 24-h ambulatory BP monitoring (ABPM), are related to increased risk of organ damage and cardiovascular (CV) events [4]. The physiological circadian pattern presents a slow BP rise soon before awakening, frequently followed by two peaks occurring in the morning and between afternoon and early evening, respectively. Moreover, a nadir is detectable during late midday, whereas the mean systolic BP value is expected to decline by 10 to 20% during night-time with respect to diurnal hours (dipper pattern). The nocturnal BP lowering is mainly attributable to a reduced activity of the sympathetic nervous system (SNS), associated with relevant decreases in monoamine levels, heart rate and stroke volume, as well as in total peripheral vascular resistance. A defective function of SNS-mediated regulation of BP can be responsible for pathological nocturnal dipping patterns, mainly consisting of less than 10% reduction with respect to diurnal BP values (non-dipper pattern) [5]. Dipper pattern abnormalities may also include higher than 20% decrements in night-time BP values (extreme dipper pattern), or even nocturnal BP increases (reverse dipper pattern) [5]. A pathological dipping pattern is frequently observed in secondary or refractory hypertension, and also in other diseases such as type 1 or 2 diabetes mellitus (T2DM), chronic kidney disease, autonomic dysfunction, or sleep apnea syndromes [6, 7]. In particular, insulin resistance (IR) and hyperinsulinemia are correlated with arterial hypertension and altered dipping features driven by multiple mechanisms including [8]: (1) enhanced catecholamine synthesis [9]; (2) exaggerated RAAS activity leading to an excessive sodium resorption [10]; (3) decreased peripheral production of nitric oxide (NO), associated with an increased peripheral vascular resistance [11, 12].

Previous studies demonstrated that patients with 1-h post-load plasma glucose ≥ 155 mg/dl, during an oral glucose tolerance test (OGTT), present an increased risk to develop T2DM, among subjects with normal glucose tolerance (NGT) [13]. In addition, recent observations suggest that these subjects (NGT ≥ 155) are more susceptible to clinical/subclinical organ damage and CV risk than NGT patients with 1-h plasma glucose levels < 155 mg/dl (NGT < 155) [14–16]. However, it is not known whether NGT ≥ 155 subjects exhibit an alteration of the physiological BP circadian rhythm. According with this, the aim of the present study to determine whether in newly diagnosed hypertensive patients there is a correlation between blood glucose at 1 h during OGTT and the 24 h blood pressure profile.

## Methods

### Study population

We recruited 810 newly diagnosed hypertensive Caucasian patients (448 men and 362 women, mean age 49.2 ± 8.9 years), referring to the Internal Medicine and Geriatrics Department of “Magna Graecia” University Hospital of Catanzaro, included in the “CAtanzaro MEtabolic RIsk factors Study” (CATAMERIS) [17]. The time interval of enrollment was from January 2018 to December 2022. The enrolled patients had never taken antihypertensive drug therapy. All participants underwent a complete physical examination, anthropometric evaluation with measurement of weight and height, and calculation of the body mass index (BMI). Laboratory tests, BP measurement and 24-h blood pressure monitoring were also carried out. Causes of secondary hypertension were excluded by appropriate clinical and biochemical tests. Other exclusion criteria were history of malignancies, alcohol or drug addiction, use of glucoactive drugs, CV diseases (ischemic heart diseases, valvular heart diseases, congestive heart failure, peripheral vascular diseases), gastrointestinal disorders (inflammatory bowel disease associated with malabsorption, chronic pancreatitis, liver failure), chronic renal failure, and metabolic disorders (i.e., hyperlipidemia). The local ethics committee approved the protocol, and informed written consent was obtained from all participants (code protocol number 2012.63).

### Blood pressure measurement

BP values were obtained according to current guideline recommendations (1). BP measurement was performed after a rest of at least 5 min, on the non-dominant arm of patients in the sitting position, using a semi-automatic sphygmomanometer (OMRON, M7 Intelli IT). Patients underwent three separate BP measurements, on three different occasions, at intervals of at least 2 weeks. BP values ​​were obtained by averaging at least two of the three measurements, carried out at 2-min intervals. In our investigation, arterial hypertension was defined using the European Society of Hypertension (ESH) and the European Society of Cardiology (ESC) criteria as systolic BP (SBP) ≥ 140 mmHg and/or diastolic BP ≥ 90 mmHg [1].

### Laboratory measurements

All laboratory measurements were performed after a fast of at least 12 h. A 75 g OGTT was performed sampling plasma glucose and insulin levels at baseline and after 30, 60, 90, and 120 min. Glucose tolerance status was defined on the basis of OGTT according to the World Health Organization (WHO) criteria. Plasma glucose levels were assessed by the glucose oxidation method (Beckman Glucose Analyzer II; Beckman Instruments, Milan, Italy), and plasma insulin concentrations were measured by a chemiluminescence-based assay (Roche Diagnostics). Type 2 diabetes mellitus (T2DM) was defined according to the American Diabetes Association (ADA) criteria [18]. The categories of glucose homeostasis were defined based on ADA criteria, NGT were defined as fasting plasma glucose (FPG) < 5.5 mmol/L (99 mg/dl) and 2-h plasma glucose concentration < 7.8 momol/L (140 mg/dl), IGT as FPG < 6.1 mmol/L (110 mg/dl) and 2-h plasma concentration 7.8–11.1 mmol/L (140–200 mg/dl), T2DM as FPG ≥ 7 mmol/L and 2-h plasma concentration ≥ 11.1 mmol/L (220 mg/dl). Insulin sensitivity was evaluated using the Matsuda index (insulin sensitivity index, ISI) calculated as follows: 10.000/square root of [fasting glucose (millimoles per liter) × fasting insulin (milliunits per liter)] × [mean glucose3meaninsulin during OGTT] [19]. Triglycerides, total, low-density lipoprotein (LDL), and high-density lipoprotein (HDL) cholesterol concentrations were determined by enzymatic methods (Roche Diagnostics, Mannheim, Germany). Estimated glomerular filtration rate (e-GFR) values were calculated by using the equation proposed by investigators in the chronic kidney disease epidemiology (CKD-EPI) collaboration [20].

### Clinic blood pressure monitoring

The 24-h ABPM was performed by means of oscillometric methodology using the SpaceLabs 90207 monitors (SpaceLabs, Inc, Redmond, WA). On the basis of dipping status definitions, dipper partner characterized the reduction of the mean systolic BP value by 10 to 20% during night-time with respect to diurnal hours, non-dipper partner mainly characterizes a reduction less than 10% with respect to diurnal BP values, extreme dipper partner defines the decrements in night-time BP values higher than 20% and reverse dipper partner characterized nocturnal BP increases. During BP recording, patients maintained regular physical activity and a regular sleep–wake rhythm, sleeping from 10:00 pm to 7:00 am. BP measurements took place every 15 min during the day (7:00 am–10:00 pm) and every 30 min during the night (10:00 pm–7:00 am). The analysis of collected data allowed classifying patients based on nocturnal BP profiles into four categories including dippers, non-dippers, extreme dippers, and reverse dippers. In particular, BP drop was calculated using the following formula: (diurnal SBP − nocturnal SBP)/(diurnal SBP × 100) [5]. Dipping status was calculated based on systolic BP

### Statistical methods

Data are expressed as mean ± standard deviation (SD). All comparisons were performed using SPSS 20.0 statistical software for Windows (SPPS, Inc., Chicago, IL, USA). ANOVA test was used to evaluate differences regarding clinical and biological parameters referring to the different patient groups. Post hoc Bonferroni analysis was performed for multiple comparisons. The *χ*^2^ test was used to analyze nominal data. A linear regression analysis was made in the whole population in order to correlate nocturnal BP drop with multiple covariates, such as age, BMI, LDL cholesterol, uric acid, e-GFR, high-sensitivity C-reactive protein (hs-CRP), fasting glucose, 1-h glucose, 2-h glucose, fasting insulin, 1-h insulin, 2-h insulin and Matsuda index. Subsequently, variables that reached statistical significance were entered into a stepwise multiple regression model, with the aim of investigating the magnitude of their effect on nocturnal BP drop.

Finally, a logistic regression analysis was carried out considering the pathological nocturnal BP drop (non-dipper and reverse dipper) as dependent variable. In this analysis, 1-h post-load glucose was considered as a dichotomous variable, excluding diabetic patients, and adjusting statistical model for age, sex, BMI, smoking, e-GFR, uric acid, hs-CRP and Matsuda index, thus assessing odds ratio (OR) and 95% confidence interval (95% CI). The statistical differences were considered significant for *p* value < 0.05.

## Results

### Study population

Table 1 shows the demographic, clinical and biochemical characteristics of the entire study population consisting in 810 patients. According to OGTT, the overall population was divided into four groups: 344 NGT < 155 (42.5%), 187 NGT ≥ 155 (23.1%), 201 impaired glucose tolerance (IGT) patients (24.8%) and 78 T2DM patients (9.6%). There was no significant difference among the four groups regarding gender, age, and total cholesterol. In contrast, among groups, there was a progressive increase in fasting plasma glucose levels (*p* < 0.0001), 1-h post-load plasma glucose levels (*p* < 0.0001), 2-h post-load plasma glucose levels (*p* < 0.0001), fasting insulin (*p* < 0.0001), 1-h insulin (*p* < 0.0001), 2-h insulin (*p* < 0.0001), triglyceride concentrations (*p* = 0.017), and hs-CRP (*p* = 0.001). In addition, e-GFR showed significant and progressive impairment across the four groups (*p* < 0.0001), the same pattern was observed for HDL concentrations (*p* = 0.012) and Matsuda index (*p* < 0.0001). From Bonferroni post hoc test, NGT ≥ 155 patients showed higher values of fasting insulin (*p* = 0.004), 1-h insulin (*p* < 0.0001), 2-h insulin (*p* < 0.0001), compared to NGT < 155 subjects. In addition, in comparison with NGT < 155 patients, NGT ≥ 155 exhibited higher levels of hs-CRP (*p* = 0.001) and decreased values of Matsuda index (*p* < 0.0001) and e-GFR (*p* = 0.001). No statistically significant differences were observed in the comparison between NGT ≥ 155 and IGT.

**Table 1:** Demographic, clinical and biochemical characteristics of the study population according to the state of glucose tolerance

	All	NGT < 155	NGT ≥ 155	IGT	T2DM	*p*
	(*n* = 810)	(*n* = 344)	(*n* = 187)	(*n* = 201)	(*n* = 78)	
Sex, m/f	379/321	158/153	95/66	96/75	30/27	0.479
Age, years	49.2 ± 8.9	48.5 ± 10.5	49.7 ± 8.2	49.9 ± 6.8	49.6 ± 6.9	0.295
Smokers, *n* (%)	148 (21.14)	77 (21.8)	40 (24.8)	24 (14)	7 (12.3)	0.011
Waist circumference, cm	100.4 ± 10.6	98.5 ± 11.1	100.7 ± 9.7	102.9 ± 10.6	102.3 ± 8.9	< 0.0001
BMI, Kg/m^2^	29.1 ± 3.9	28.7 ± 4.2	29.5 ± 4.1	29.5 ± 3.5	29.1 ± 3.1	0.031
Fasting glucose, mg/dl	97.5 ± 15.1	90.8 ± 9.7	97.1 ± 12.5	101.3 ± 13.5	118.3 ± 21.1	< 0.0001
1-h glucose, mg/dl	165.7 ± 51.9	120.1 ± 23.1	189.9 ± 33.3	191.8 ± 34.7	241.3 ± 43.6	< 0.0001
2-h glucose, mg/dl	131.8 ± 49.1	102.7 ± 21.0	115.9 ± 21.2	166.0 ± 17.4	240.6 ± 46.2	< 0.0001
Fasting insulin, μU/ml	13.4 ± 7.2	11.2 ± 6.2	13.4 ± 6.4	15.8 ± 7.8	17.0 ± 7.9	< 0.0001
1-h insulin, μU/ml	100.2 ± 62.6	75.1 ± 51.3	131.7 ± 73.9	111.7 ± 54.1	106.1 ± 55.1	< 0.0001
2-h insulin, μU/ml	91.8 ± 66.9	59.0 ± 46.3	95.7 ± 58.4	132.3 ± 76.3	123.2 ± 63.1	< 0.0001
Matsuda index	64.9 ± 37.9	88.1 ± 38.8	55.1 ± 30.5	44.4 ± 22.3	36.2 ± 14.9	< 0.0001
Total cholesterol, mg/dl	201.7 ± 39.5	203.7 ± 36.8	208.9 ± 36.9	198.3 ± 42.3	197.7 ± 47.3	0.967
HDL, mg/dl	50.0 ± 13.6	52.1 ± 14.3	49.3 ± 13.9	48.4 ± 12.2	46.7 ± 12.2	0.012
Triglycerides, mg/dl	134.8 ± 62.7	125.7 ± 60.6	132.4 ± 59.8	141.5 ± 65.4	162.4 ± 61.5	0.017
Hs-CRP, mg/l	3.2 ± 2.2	2.8 ± 2.1	3.2 ± 2.2	3.6 ± 2.4	4.1 ± 2.2	0.001
e-GFR, ml/min/1.73 m^2^	100.8 ± 24.2	106.7 ± 25.4	97.4 ± 22.7	96.7 ± 23.6	94.8 ± 21.6	< 0.0001

*NGT* normal glucose tolerance, *IGT* impaired glucose tolerance, *T2DM* type 2 diabetes mellitus, *BMI* body mass index, *ISI* insulin sensitivity index, *hs-CRP* high-sensitivity C-reactive protein, *e-GFR* estimate glomerular filtrate rate

### Hemodynamic and ABPM parameters

**Fig. 1:**
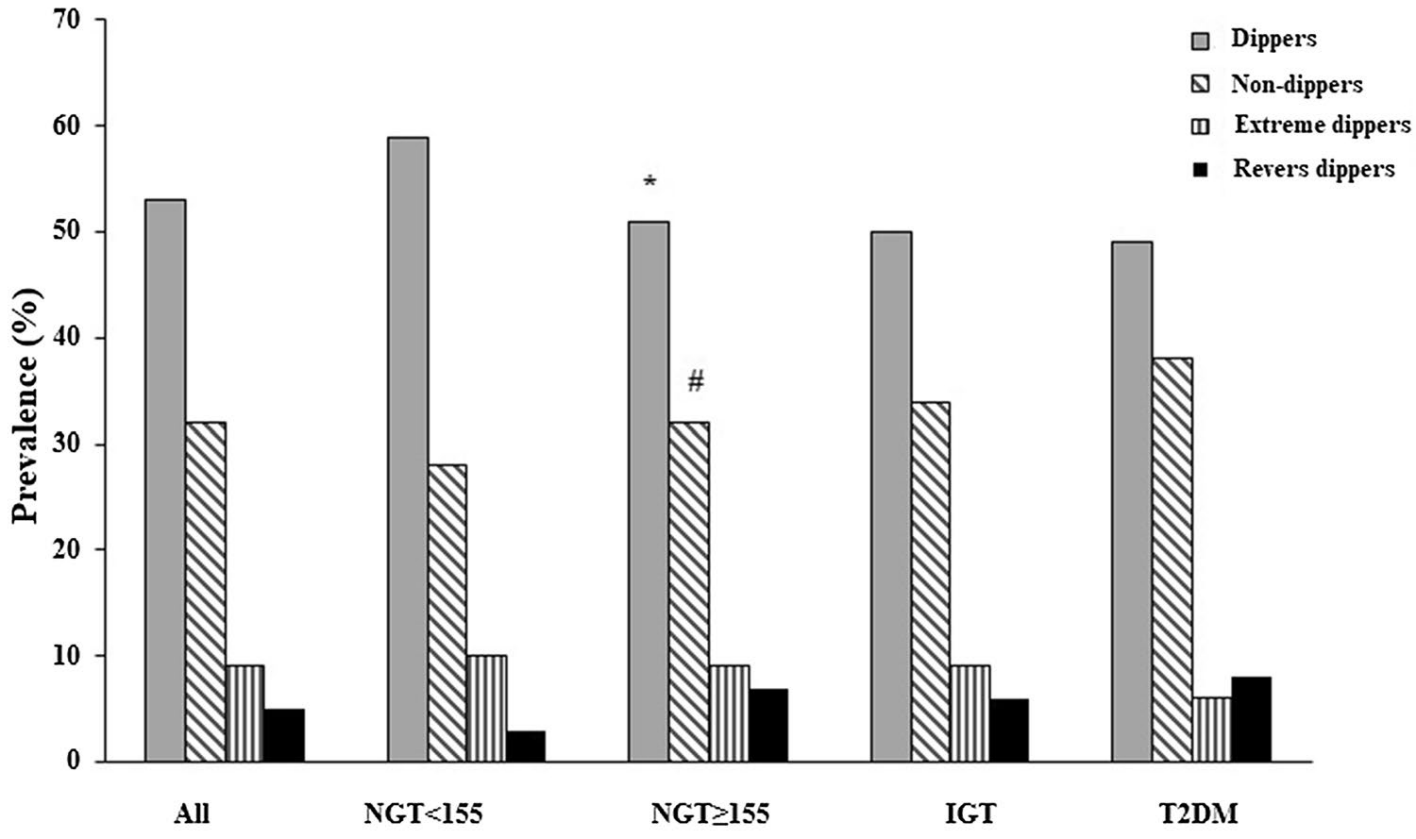
Prevalence of nocturnal blood pressure drop in the whole study population and according to the state of glucose tolerance. *p = 0.017, ^#^*p* = 0.001. *NGT* normal glucose tolerance, *IGT* impaired glucose tolerance, *T2DM* type 2 diabetes mellitus

Table 2 summarizes clinical, hemodynamic and ABPM parameters of the entire study population. Throughout the four study groups, and in regard to all evaluated variables, there were no statistically significant differences, except for the mean 24-h SBP, which reached the highest value in the T2DM group (*p* = 0.009). In addition, from Bonferroni post hoc test, there was no statistically significant differences between NGT ≥ 155 vs IGT, regarding 24-h SBP values. Considering the dipping pattern, the proportion of non-dippers in NGT ≥ 155 (36.4%) was higher than NGT < 155 (29.6%) and IGT (34.8%), but lower than T2DM group (52.6%) (*p* = 0.001). The proportion of dippers was lower in NGT ≥ 155 (47%) and T2DM group (34.6%), when compared with NGT < 155 (53.8%) and IGT (51.2%) (*p* = 0.017). From *χ*^2^ test, the proportion of dipper patterns in NGT < 155 was significantly higher than T2DM group (*p* = 0.002), moreover, the proportion of non-dipper patterns in NGT < 155 subjects was significantly reduced than T2DM patients (*p* = 0.001) There was no significant difference in the proportion of extreme and reverse dippers across the four groups (Fig. 1).

**Table 2:** Hemodynamic, clinical and monitored characteristics of the study population, according to the state of glucose tolerance

	All	NGT < 155	NGT ≥ 155	IGT	T2DM	*p*
	(*N* = 810)	(*N* = 344)	(*N* = 187)	(*N* = 201)	(*N* = 78)	
Clinic SBP, mmHg	142.8 ± 13.1	141.3 ± 13.1	144.0 ± 12.6	143.7 ± 12.3	144.4 ± 11.1	0.054
Clinic DBP, mmHg	89.7.4 ± 8.1	89.6 ± 7.7	90.9 ± 6.4	89.3 ± 9.1	89.3 ± 9.7	0.086
Clinic HR, bpm	72.1 ± 10.7	71.1 ± 10.5	73.1 ± 10.3	72.9 ± 11.2	72.8 ± 10.6	0.098
24 h-ABPM SBP, mmHg	136.4 ± 9.8	136.6 ± 10.1	135.7 ± 10.0	135.3 ± 9.0	139.4 ± 10.0	0.009
24 h-ABPM DBP, mmHg	84.1 ± 11.8	83.7 ± 12.1	83.6 ± 12.2	83.0 ± 11.0	85.1 ± 12.7	0.608
24 h-ABPM HR, bpm	71.2 ± 9.7	70.2 ± 9.7	72.7 ± 9.7	71.4 ± 10.0	72.0 ± 9.2	0.046
Diurnal ABPM SBP, mmHg	139.5 ± 9.1	139.1 ± 8.1	138.9 ± 10.2	139.0 ± 10.6	139.1 ± 10.0	0.992
Diurnal ABPM DBP, mmHg	86.2 ± 11.3	85.6 ± 11.7	85.6 ± 11.5	85.4 ± 10.5	87.2 ± 12.2	0.689
Diurnal ABPM HR, bpm	75.0 ± 9.1	74.2 ± 9.0	76.3 ± 9.2	75.3 ± 9.3	75.5 ± 8.5	0.102
Nocturnal ABPM SBP, mmHg	126.4 ± 10.9	126.2 ± 11.6	126.4 ± 12.7	126.3 ± 13.6	128.1 ± 10.3	0.668
Nocturnal ABPM DBP, mmHg	74.8 ± 10.8	74.6 ± 11.4	75.1 ± 10.8	74.4 ± 9.7	76.6 ± 11.8	0.435
Nocturnal ABPM HR, bpm	65.9 ± 8.8	65.1 ± 8.7	67.1 ± 8.8	66.2 ± 9.1	66.3 ± 7.6	0.095
Nocturnal BP drop, mmHg	7.6 ± 6.5	9.3 ± 7.1	8.4 ± 6.9	9.0 ± 6.9	7.6 ± 6.5	0.209
Extreme dippers, *n* (%)	64 (7.9)	32 (9.3)	14 (7.5)	15 (7.5)	3 (3.8)	0.425
Dippers, *n* (%)	403 (49.8)	185 (53.8)	88 (47.0)	103 (51.2)	27 (34.6)	0.017
Non-dippers, *n* (%)	281 (34.7)	102 (29.6)	68 (36.4)	70 (34.8)	41 (52.6)	0.001
Reverse dippers, *n* (%)	62 (7.6)	25 (7.3)	17 (9.1)	13 (6.5)	7 (9.0)	0.750

*NGT* normal glucose tolerance, *IGT* impaired glucose tolerance, *T2DM* type 2 diabetes mellitus, *SBP* systolic blood pressure, *DBP* diastolic blood pressure, *HR* hearth rate, *ABPM* ambulatory blood pressure monitoring

### Correlation analyses

Table 3 shows the results of the linear regression analysis, performed to evaluate the eventual association between nocturnal BP drop and several covariates. In the entire study population, nocturnal BP decline was inversely correlated 1-h glucose (*r* = − 0.261, *p* < 0.0001), 2-h glucose (*r* = − 0.122, *p* < 0.0001), 1-h insulin (*r* = − 0.204, *p* < 0.0001), 2-h insulin (*r* = − 0.145, *p* < 0.0001), high-sensitivity C-reactive protein (hs-CRP) (*r* = − 0.170, *p* < 0.0001), age (*r* = − 0.097, *p* = 0.003). Conversely, nocturnal BP decline resulted to be linked by a significant direct correlation with Matsuda index (*r* = 0.108, *p* = 0.001) and LDL cholesterol (*r* = 0.079, *p* = 0.013).

**Table 3:** Linear regression analysis between nocturnal blood pressure drop and different covariates, such as age, BMI, LDL, uric acid, e-GFR, hs-CRP, fasting glucose, 1-h glucose, 2-h glucose, fasting insulin, 1-h insulin, 2-h insulin and Matsuda index, in the entire study population

	All	
	(*N* = 810)	
	*R*	*p*
Age, years	− 0.097	0.003
BMI, kg/m^2^	− 0.043	0.110
LDL, mg/dl	− 0.079	0.013
Uric acid, mg/dl	− 0.064	0.036
e-GFR, ml/min/1.73m^2^	0.100	0.002
hs-CRP, mg/dl	− 0.170	< 0.0001
Fasting glucose, mg/dl	0.011	0.376
1-h glucose, mg/dl	− 0.261	< 0.0001
2-h glucose, mg/dl	− 0.122	< 0.0001
Fasting insulin, µU/ml	− 0.034	0.167
1-h insulin, µU/ml	− 0.204	< 0.0001
2-h insulin, µU/ml	− 0.145	< 0.0001
Matsuda index	0.108	0.001

*NGT* normal glucose tolerance, *IGT* impaired glucose tolerance, *T2DM* type 2 diabetes mellitus, *BMI* body mass index, *hs-CRP* high-sensitivity C-reactive protein, *e-GFR* estimate glomerular filtrate rate, *LDL* LDL cholesterol

In order to evaluate the predictors of nocturnal BP drop, the variables that reached statistical significance in the linear regression analysis were used for a stepwise multiple regression model (Table 4). In the overall population, the major predictor of nocturnal BP reduction was 1-h glucose which accounted for 6.7% (*p* < 0.0001) of its variation; FPG and hs-CRP contributed for 2.0% (*p* < 0.0001) and 1.9% (*p* = 0.011), respectively. Finally, a logistic regression analysis was carried out on the overall study population with the exception of diabetic patients, who were excluded in order to avoid selection bias (Table 5). This analysis was performed including the pathological nocturnal BP drop (both non-dipper and reverse dipper pattern) as dependent variable and considering one-hour post-load glucose as dichotomic value (< 155 and ≥ 155 mg/dl), adjusting the model for age, gender, BMI, smoking, e-GFR, uric acid, hs-CRP and Matsuda index. One-hour post-load glucose ≥ 155 mg/dl increased the odds of pathological nocturnal BP drop by 74%, (OR = 1.740, 95% CI 1.254–2.415, *p* < 0.0001). In addition, male gender was protective for pathological nocturnal BP drop reducing the odds by 29.4% (OR = 0.706, 95% CI 0.509–0.981).

**Table 4:** Stepwise multiple regression analysis of nocturnal blood pressure drop as a dependent variable in the entire study population

	*R*^2^ partial	*R*^2^ total	Unstandardized Beta	Standardized Beta	*P*
All (*n* = 810)					
1-h glucose, mg/dl	6.7%	6.7%	− 0.042	− 0.305	< 0.0001
Fasting glucose, mg/dl	2.0%	8.7%	0.089	0.187	< 0.0001
Hs-CRP	1.9%	10.6%	− 0.267	− 0.086	< 0.0001

*NGT* normal glucose tolerance, *IGT* impaired glucose tolerance, *T2DM* type 2 diabetes mellitus, *hs-CRP* high-sensitivity C-reactive protein, *e-GFR* estimate glomerular filtrate rate, *LDL* LDL cholesterol

**Table 5:** Logistic regression analysis between 1-h binary glucose and pathological nocturnal blood pressure drop in the whole population (excluding diabetics), analysis adjusted for age, gender, body mass index, smoking, uric acid, glomerular filtrate, high-sensitivity C-reactive protein and Matsuda index

		OR	*p*	95% CI
1-h glucose, mg/dl	Yes/no	1.740	< 0.0001	1.254–2.415
Fasting glucose, mg/dl	1 mg/dl	0.987	0.116	0.970–1.003
Sex	Male/female	0.891	0.543	0.615–1.291
Age, years	1 year	1.020	0.029	1.002–1.038
Smoke	Yes/no	1.027	0.519	0.779–1.640
BMI, Kg/m^2^	1 kg/m^2^	0.988	0.599	0.945–1.033
Uric acid, mg/dl	1 mg/dl	1.055	0.478	0.910–1.223
e-GFR, ml/min/1.73 m^2^	1 ml/min/1.73 m^2^	0.991	0.020	0.984–0.999
hs-CRP, mg/dl	1 mg/dl	1.068	0.103	0.987–1.157
Matsuda index	1 unità	0.965	< 0.0001	0.958–0.971

*OR* odds ratio, *CI* confidence interval, *BMI* body mass index, *e-GFR* estimated glomerular filtrate rate, *hs-CRP* high-sensitivity C-reactive protein

## Discussion

Our study confirms the existence of an association between altered glucose metabolisms and reduced physiological BP drop at night; in fact, acquired data showed a significant reduction in the number of dippers and a significant increase in non-dippers, with the worsening of metabolic status. However, from our study, the most important finding emerges from the linear regression analysis, in fact in the whole study population, 1-h glucose was the main predictor of BP drop, justifying 6.7% of its variation.

Moreover, through a logistic regression analysis between 1-h post-load glucose (expressed as a dichotomous value) and the pathological nocturnal BP drop, we demonstrated that glucose values ≥ 155 mg/dl at 1-h post-load increased the risk of a pathological nocturnal BP drop by 74%. Thus, 1-h glucose ≥ 155 in NGT subjects is associated with a high risk of having a pathological BP profile with reduced physiological nocturnal decline.

The relationship between impaired glucose metabolism and pathological dipper pattern is already known. The effects of hyperinsulinemia on SNS are well described, mainly consisting of an enhanced norepinephrine production, with consequent increases in hearth rate and systolic BP [21]. Autonomic nervous system (ANS) is able to directly regulate blood glucose levels; the parasympathetic nervous system (PNS) stimulate pancreatic beta-cells to enhance insulin secretion and facilitates enhanced tissue glucose uptake, in contrast, SNS reduces insulin secretion leading to an increase of blood glucose levels [22]. Therefore, an impairment in ANS is associated with impaired beta-cells function and alterations in cholinergic muscarinic receptor (mAChR) activities. Based on this, a study conducted in a murine model demonstrated that therapy with metformin could be used to develop new therapeutic strategies that normalize ANS impairment observed in metabolic disease [22]. In non-dipper patients, several reports showed during the nocturnal hours that both a reduced activation of parasympathetic nervous system and the persistence of SNS activity significantly contributed to the lack of nocturnal BP drop [23, 24]. Data about the roles of hyperinsulinemia and IR in nocturnal dipping status are controversial. Even if in our study, we demonstrated a significant correlation between Matsuda index, 1-h and 2-h insulin and the nocturnal BP drop, another investigation did not demonstrate significant correlations between IR and pathological dipping status [25]. However, literature data quite consistently show that diabetic patients are characterized by the highest prevalence of abnormal dipping patterns. In a cross-sectional study involved 12.765 hypertensive patients from the Hygia Project, the diabetic group was more likely to experience subclinical and clinical organ damage, higher systolic BP during night-time and higher prevalence of non-dipper pattern [26]. After multivariate logistic regression, reverse-dipper and non-dipper BP patterns were found to be correlated with T2DM, when compared with dipper pattern. In addition, fasting glucose was negatively correlated with the nocturnal BP drop. These findings are consistent with our results, as shown by the higher prevalence of pathological dipping profile in patients with diabetes and altered glucose metabolism. Based on these observations, it can be reasonably argued that patients with an apparent healthy metabolism can hide pathological BPV. Conversely, patients with pathological dipping status need an accurate metabolic screening, in order to discover an unrecognized altered glucose metabolism. The clinical importance of finding out and further analyzing NGT ≥ 155 patients by means of ABPM derives from the large evidence of subclinical organ damage in NGT ≥ 155 and of increased major adverse cardiac event (MACE) risk in patients with pathological dipping status [27–30].

A consistent bulk of evidence established a correlation between pathological dipping status and subclinical/clinical organ damage. In a cohort of hypertensive patients, echocardiographic evaluation showed that left ventricular hypertrophy rates progressively increased from extreme dippers to dippers, non-dippers and reverse dippers (5%, 9%, 17% and 31%, respectively) [31]. In addition, left ventricular mass index (LVMI) was higher in non-dippers and reverse dippers, when compared to dippers [32]. Furthermore, several clinical investigations such as the AASK trial [33] and the APRODITE study [34] reported a higher prevalence of proteinuria in reverse dippers compared to non-dippers, dippers and extreme dippers. A large European investigation, which recruited 2800 drug-naïve hypertensive patients, showed that microalbuminuria reached the highest value in reverse dippers [35]. Further data provided a strong evidence in support of the correlations existing between altered nocturnal dipping patterns and subclinical/clinical vascular dysfunctions. In particular, when compared with dippers and extreme dippers, reverse dippers and non-dippers were shown to have a greater thickness of intima-media layers of common carotid artery [36]. Moreover, the reverse dipper pattern was a strong predictor of the development of mild carotid plaques [37]. Other authors investigated the relationship between pathological nocturnal dipping status and MACE. In this regard, during a median follow-up of 4.2 years, an interesting study evaluated the CV and renal outcomes of 436 patients affected by chronic kidney disease and arterial hypertension, stratified according to BP quintiles and nocturnal dipping status. The results showed an increased risk of CV and renal events in the highest day-time and night-time systolic and diastolic BP quintiles. Moreover, non-dipper patients had a HR of 1.95 (CI 1.15–3.31) for CV events, and a HR of 1.62 (1.08–2.44) for renal death, whilst reverse dippers had a HR of 2.11 (CI 1.11–4) for CV events and a HR of 1.72 (1.04–2.85) for renal death, respectively [38]. The relationship between pathological dipping status and increased risk of cerebrovascular events was detected by a Japanese study that enrolled 575 elderly patients affected by arterial hypertension and subclassified according to the dipping status. After an average follow-up per patient of 41 months, the study found a J-shaped relationship between dipping pattern and stroke, considering that reverse dippers and extreme dippers manifested more events than dippers and non-dippers. The same trend was confirmed by brain magnetic resonance, which revealed that the percentage of silent lacunar infarcts was higher in extreme dippers and in reverse dippers. Furthermore, a higher number of haemorrhagic strokes was reported in reverse dippers, while a higher number of ischemic strokes was found in extreme dippers [39]. The above observations include only a portion of the robust evidence which has convincingly established a correlation between pathological dipping status and increased risk of silent and clinical cerebrovascular disorders [40–42], coronary artery diseases [43], and heart failure [44, 45].

In comparison to NGT < 155 group, subjects classified as NGT ≥ 155 are characterized by a worse glucidic, lipidic, and inflammatory patterns, as well as by a worse renal function, thus showing similarities with the profiles of other patients with metabolic dysfunction, such as IGT and T2DM. NGT ≥ 155 patients frequently presented abnormal dipping patterns, probably due to pathological interactions occurring between hyperinsulinemia/hyperglycemia and SNS persistent activation, as shown by linear and multivariate regression models. Based on the strong relationships existing among metabolic disorders, pathological BPV and burden of subclinical/clinical diseases, it could be useful to screen patients with altered glucose metabolism by means of a 24-h ABPM. Finally, it might be also important to rule out that patients with pathological dipping patterns can eventually suffer from previously unrecognized dysfunctions of glucose metabolism. However, the present study has some limitations. One of the limitations is the lack of data about sleep quantity and quality, moreover dipping status calculated based on selected fixed time intervals has low reproducibility.

In conclusion, the major relevance of our study is to suggest that an OGTT should be performed in patients resulted non-dippers after 24-h ABPM, to better stratify cardio metabolic risk. In addition, it is possible in future, to speculate that 1-h post-load during OGTT in pregnant women could identify those at risk of preeclampsia.

## Data Availability

Not available.
